# Management of post-traumatic craniovertebral junction dislocation: A PRISMA-compliant systematic review and meta-analysis of casereports

**DOI:** 10.1007/s10143-020-01366-4

**Published:** 2020-08-14

**Authors:** Tomasz Klepinowski, Bartosz Limanówka, Leszek Sagan

**Affiliations:** Department of Neurosurgery, Pomeranian Medical University Hospital No. 1, Szczecin, Poland

**Keywords:** Craniovertebral junction dislocation, Trauma, Atlantoaxial joint, Atlantooccipital joint

## Abstract

Although historically considered fatal, with the advent of improved pre-hospital care, traumatic dislocation of the craniovertebral junction (CVJ) has been increasing in prevalence in neurosurgical centers. As more survivors are reported each year, a timely review with meta-analysis of their management seems necessary. PRISMA checklist was followed step by step. PubMed and Web of Science databases were searched using words “craniovertebral junction dislocation” and their corresponding synonyms. Study eligibility criteria included research studies from 2015 onwards that delineated adult and pediatric patients with confirmed post-traumatic atlantooccipital dislocation (AOD) or atlantoaxial dislocation (AAD) who survived until proper treatment. Of 1475 initial records, 46 articles met eligibility criteria with a total of 141 patients with traumatic CVJ dislocation. Of the patients, 90 were male (63.8%). Mean age of the cohort was 33.3 years (range 1–99 years). Trauma that most often led to this injury was road traffic accident (70.9%) followed by falls (24.6%). The majority of authors support posterior instrumentation of C1-C2 (45.2%) especially by means of Goel-Harms method. At mean follow-up of 15.4 months (range 0.5–60 months), 27.2% of treated patients remained neurologically intact. Of initially symptomatic, 59% improved, 37% were stable, and 4% deteriorated. Instrumenting the occiput in cases of pure AAD was associated with lower chance of neurological improvement in chi-square test (*p* = 0.0013) as well as in multiple linear regression (*β* = − 0.3; *p* = 0.023). The Goel-Harms C1-C2 fusion is currently the most frequently employed treatment. Many survivors remain with no deficits or improve, rarely deteriorate. Involving the occiput in stabilization in cases of AAD without AOD might be related with worse neurological prognosis.

**Electronic supplementary material:**

The online version of this article (10.1007/s10143-020-01366-4) contains supplementary material, which is available to authorized users.

## Introduction

Craniovertebral junction (CVJ) is a term encompassing occipitoatlantal and atlantoaxial complex of joints and ligaments. The former of the joints is said to provide stability, whereas the latter is responsible for the wide range of motion, accounting for more than half of the cervical spine rotation [1–3]. Dislocation of the junction happens when the integrity of the articular capsule is breached and might occur at each of the joints. Furthermore, it may be of congenital, inflammatory, or traumatic etiology. Traumatic dislocation of craniovertebral junction, although once considered fatal, has been proven numerous times that it can be a survivable injury [4]. It is mainly due to improvement made in the pre-hospital stage of care. Therefore, more and more survivors are encountered who require proper stabilization, which can be either conservative or surgical. Traumatic atlantooccipital dislocation (AOD) constitutes 1% of all injuries to the cervical spine and is believed to be less common than traumatic atlantoaxial dislocation (AAD) which constitutes 2.7% [5].

Rationalizing the study, we found no systematic review on the post-traumatic CVJ dislocation. Hence, considering recent advances in resuscitation, radiological diagnostic tools, and increasing choice of neurosurgical options, a timely review seems necessary. Utilizing the structured guidelines of the PRISMA (Preferred Reporting Items for Systematic Reviews and Meta-Analyses) group has become a standard for high-quality review studies. As proven by Sampayo-Cordero et al. in 2018 [6], a meta-analysis of case reports is accurate and agrees with a meta-analysis of clinical randomized studies and is dedicated for rare diseases that often times have zero events in either both arms. Since adequate records of post-traumatic CVJ are reported solely in case reports or case series, a meta-analysis based on case reports is well rationalized. This study aims to answer questions of what state-of-the-art treatment patients with traumatic dislocation of CVJ are currently receiving, what outcomes they are likely to obtain, and whether plating the occiput affects patients’ outcome.

## Materials and methods

PRISMA checklist was followed step by step (Electronic Supplementary Material No. 1). Participants, interventions, context, outcome, and study designs are shown in Table 1. On 17 February 2020, two databases, PubMed Medline and Web of Science, were perused using search words “craniovertebral junction dislocation” and their corresponding synonyms. Full electronic search strategy can be found here (Electronic Supplementary Material No. 2).

**Table 1:** PICOS acronym describing characteristics of this review study

Acronym	Definition	This study
P	Participants	Children and adults with post-traumatic AOD, AAD, or both who survived until treatment
I	Intervention	Anterior or posterior reduction and instrumentation with levels of fusion and status of occipital involvement. If there was no surgery, conservative management details of traction and bracing would be presented
C	Context	Trauma—its mechanism as well as basic demographic data of patients
O	Outcome	Neck pain, NDI, JOA, mJOA, Nurick scale, ASIA, subjective improvement, fusion rates
S	Study designs	Cross-sectional and longitudinal cohort studies, case series (*n* ≥ 3), case reports (*n* ≤ 2)

AAD, atlantoaxial dislocation; AOD, atlantooccipital dislocation; ASIA, American Spinal Injury Association Impairment Scale; mJOA, modified Japanese Orthopedic Association score; NDI, neck disability index

Eligibility criteria included latest research studies from 2015 to 2020 that delineated patients with post-traumatic craniovertebral junction dislocation. We defined dislocation as a ligamentous injury with ensuing misalignment of the facets. Thus, what was searched was radiological evidence of either obvious loss of facetal contact or positive testing for classic radiological markers. Concomitant lesions or fractures within the CVJ were acceptable as long as post-traumatic facetal misalignment was present. Inclusion and exclusion criteria are shown in Table 2. Extraction of the data from the gathered reports was conducted by the first author (TK) and then independently checked and confirmed by the co-authors (BL and LS) so as to ensure minimal risk of bias of individual studies. Collected variables included number of traumatic cases in a given publication, patients’ sex, age, mechanism of trauma, resultant injury, underlying disease (if present), therapeutic interventions, levels of fusion, surgical technique, and outcomes (subjective pain analysis, neurological assessment, fusion rates) at a specified follow-up period. Conservative treatment information was retrieved only when there was no subsequent surgery. If a patient received both conservative and surgical management, focus was on the latter. Pain relief as an outcome was noted only if there was pain at presentation. Thus, a documented change in pain intensity after treatment was searched for extraction. Neurological status was assessed both preoperatively and postoperatively and tendency was written down. Accepted forms of neurological status assessment were as follows: quantified scores of myelopathic grading systems such as Japanese Orthopedic Association (JOA) score, its modified variant (mJOA), American Spinal Injury Association (ASIA) score, Nurick score, as well as authors’ subjective examination. Due to lack of unified neurological assessment methods among the authors, gross tendencies were grouped into the following categories: remained intact, improved, stable, and deteriorated. Additionally, fusion rates were collected—fusion was defined as documented lack of movement of the stabilized joints assessed on lateral X-ray. Subjective authors’ statement of solid fusion was acceptable. Although subjectivity adds a risk of bias, we assumed that proper radiological assessment had been done in order to declare fusion. No funding was expected at the beginning of the study and none was obtained throughout the process. Statistical analysis of the data was performed by means of Statistica 13 (TIBCO Software Inc.). Pearson’s chi-square test would be used for categorical variables, whereas continuous variables would be computed by Wilcoxon-Mann-Whitney or Kruskal-Wallis test. Multiple linear regression was used to assess relations between neurological change at follow-up (dependent variable) and sex, age, type of dislocation, and plating the occiput (independent variables).

**Table 2:** Inclusion and exclusion criteria for the studies screened and checked for eligibility

Inclusion criteria	Exclusion criteria
1. Publications from 2015 to 2020 2. Dislocation of atlantooccipital or atlantoaxial joints 3. Trauma as the primary cause (underlying diseases and fractures are acceptable) 4. Adequate records for evaluation 5. Full text available in English or Polish 6. Treatment method listed	1. Reoperations 2. Commentaries 3. Review articles 4. Radiological articles 5. Insufficient medical history 6. Cadaveric or animal studies 7. Subject died before treatment was employed 8. Dislocation due to non-traumatic causes (congenital, inflammatory, syndromic)

## Results

**Fig. 1:**
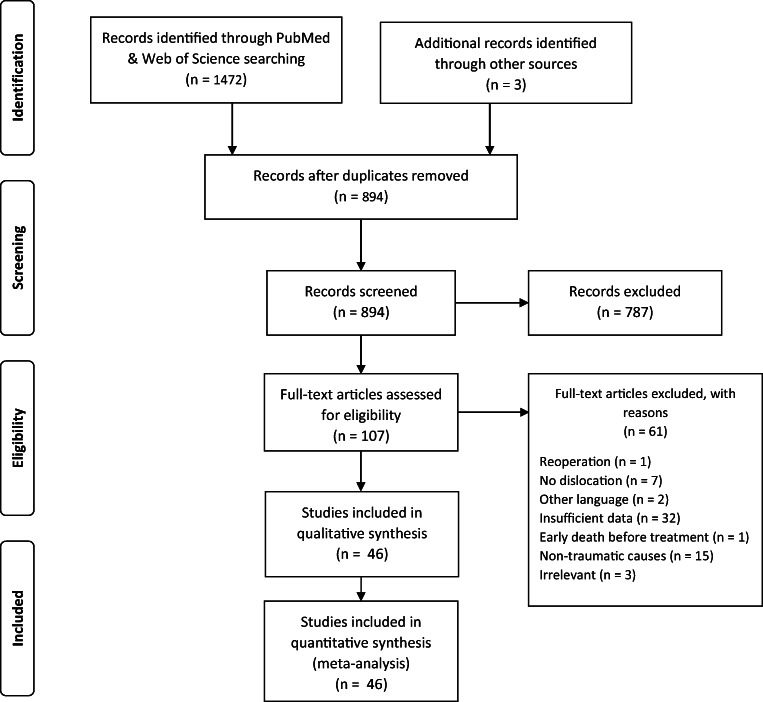
A flow diagram depicting the process of including studies into the review and meta-analysis

**Fig. 2:**
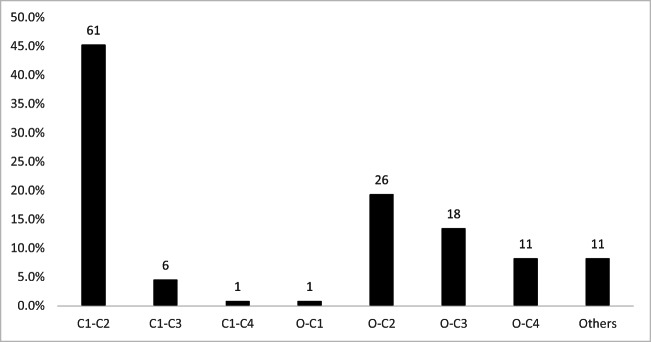
A graph showing levels of instrumentation that were addressed most frequently. A number of patients with fusion at a given level are shown above the bars

The PRISMA flow diagram shows steps of the inclusion process (Fig. 1). Initial search presented 1475 titles. Having removed duplicates, 894 titles and abstracts were collected for screening (Table 3). A total of 46 full-text articles were deemed eligible for inclusion [3, 7–50]. The highest level of evidence of the included papers was IV. They comprised 141 cases, 90 of which were male (63.8%), 46 were female (32.6%), and gender of 5 patients was not stated. Mean age of the cohort was 39.2 years (range 8 months to 99 years). For adults mean age was 46.7 years (range 19–99 years). On the other hand, in the pediatric subpopulation, mean age was 4.9 years (range 8 months to 17 years). Mechanism of injury was known in 110 patients (78.0%). Among them, the most common trauma was road traffic accident (*n* = 78) accounting for more than two-thirds of the cases (70.9%). Falls were the second leading cause of the traumatic CVJ dislocation constituting nearly a quarter of cases (24.6%). Occasionally, jumping headfirst into the water, assault, epileptic seizure, or being hit by a falling object might also lead to this condition (altogether 4.5%). Diagnosis in majority of cases was atlantoaxial dislocation (62.4%, *n* = 88), followed by atlantooccipital dislocation (27.7%, *n* = 39) and combined AAD with AOD (9.9%, *n* = 14). Most authors go for surgical management even if initial nonoperative reduction was achieved. In total, 95.7% (*n* = 135) were operated on at some point, either as a primary treatment option or as a result of failed initial conservative management. Levels that were subject of fusion most frequently were as follows: C1-C2 (45.2%, *n* = 61), O-C2 (19.3%, *n* = 26), O-C3 (13.3%, *n* = 18), O-C4 (8.1%, *n* = 11), and C1-C3 (4.4%, *n* = 6). 8.1% of the treated (*n* = 11) were either unspecified or other levels (Fig. 2). For AOD the most common level of stabilization was O-C2 (35.9%, *n* = 14). Similarly for combined AOD with AAD, O-C2 was usually fused (57.1%, *n* = 8). Choice of level differed significantly for AAD and AOD (*p* < 0.001). 4.3% (*n* = 6) of all patients were treated conservatively with no subsequent surgery. All those nonoperative cases regarded atlantoaxial dislocation, but none atlantooccipital dislocation. This, however, was not statistically significant (*p* = 0.129, chi-square test). Neurological status prior to treatment was known in 97.2% (*n* = 137) of patients. Its quantification by means of JOA, mJOA, Nurick scale, or ASIA was done in 70 cases (49.6%). The remaining was authors’ subjective examination. 27.2% (*n* = 37) were neurologically intact and remained so at follow-up, whereas 76.3% (*n* = 100) had neurological deficits on initial examination. Among the latter, 59% improved (*n* = 59), 37% were stable (*n* = 37), and 4% deteriorated (*n* = 4). Of all, 49.6% (*n* = 70) had their occipital bone involved in instrumentation. Within the group with initial neurological signs, patients without the occiput instrumentation were more likely to improve (48.1% vs 33.3%) and less likely to remain neurologically stable (9.3% vs 41.7%) (*p* = 0.0013, chi-square test; regards mainly pure AAD patients) at a mean follow-up of 15.4 months (range 0.5–60 months). Hindered improvement after plating the occiput was observed also in multiple linear regression model with the following dependent variables: mean age, sex, type of dislocation, and plating the occiput (Table 4). Debilitating neck pain was reported preoperatively by 43 patients (30.5%). Of those, at follow-up visit, neck pain improved in 36 patients (83.7%). Change in pain at a follow-up was not correlated with the status of occipital plating (*p* = 0.4091). Assessment of fusion rate was done in 81 survivors (57.4%). At follow-up visits, solid fusion was declared in 76 (93.8%).

**Table 3:** Summarized reviewed studies

Authors	Year	Cases (M/F)	Mean age (years)	Injury (*n*)	Diagnosis and concomitant anomalies (*n*)	C or S^a^	Fusion level	Technique	Mean FU (mo)	Outcome^b^ [% of patients in the series]
Grover [7]	2020	5 (3/2)	1,8	N/A	AAD; OF A-D II (3), OO (1)	S	C1-C2 (4), O-C3 (1)	SLW (4) + OP (1)	14.4	20% improved; 80% stable; 100% fusion rate
Singla [8]	2020	1 (1/0)	25	RTA	AAD rotatory Fielding I	S	C1-C2	LM-P	N/A	100% remained intact and NP reduced
Barimani [9]	2019	1 (0/1)	66	Ritual	AAD rotatory Fielding II	S	C1-C2	LM-P	3	100% remained intact and NP reduced
Biakto [10]	2019	1 (1/0)	17	RTA	AAD anterior; OF A-D II; C1 Gehweiler II	S	O-C4	OP-LM-P	2	100% improved and NP reduced
Ghailane [11]	2019	1 (1/0)	89	Fall	AAD posterior	C	None	Close reduction + rigid collar for 2 mo	8	100% remained intact and NP reduced
Garcia-Pallero [12]	2019	1 (0/1)	28	RTA	AAD rotatory	C	Nonoperative	Traction + rigid collar for 16 w	48	100% NP reduced
Tavolaro [13]	2019	2 (2/0)	44	RTA (2)	AAD (2): posterior (1), anterior (1); JF (2); OC-C1 (1); atlas assimilation (2)	S	O-C6 (1), C1-C3 (1)	OP-TLS-LM (1); LM-P (1)	N/A	50% improved; 50% deteriorated and died
Meyer [14]	2019	9 (3/6)	57,1	RTA (3), fall (6)	AAD (9), OF A-D II (5), III (1)	S	C1-C2 (7), O-C-Th or O-C (2)	LM-P (7)	N/A	N/A
Musa [15]	2019	1 (1/0)	87	Fall	AAD lateral, OF A-D II	S	C1-C2	LM-P	18	100% NP reduced; 100% fusion rate
Keen [16]	2019	15 (7/8)	3,8	RTA (15)	AOD (10), AAD (10)	S	O-C2 (12), O-C3 (2), O-C4 (1)	OP-TLS-SLW (12) + LM (3)	33	53.3% remained intact; 26.7% improved; 20% stable 100% fusion rate
Park [17]	2019	1 (0/1)	21	Fall	AAD and AOD vertical	S	O-C4	OP-LM-P	12	100% stable; 100% fusion rate
Zitouna [18]	2019	1 (1/0)	74	Fall	AAD rotatory Fielding III, OF A-D II	S	O-C2	OP-LM	N/A	100% deteriorated and stable NP
Suzuki [19]	2018	1 (0/1)	87	Fall	AOD vertical; OCF A-M I, Tuli I (1)	S	O-C2	OP-P	N/A	100% remained intact
Minyu [20]	2018	1 (1/0)	30	RTA	AAD posterolateral; OF A-D II	S	C1-C2	LM-P	60	100% remained intact and NP reduced; 100% fusion rate
Tobert [21]	2018	1 (1/0)	35	RTA	AOD vertical & AAD vertical	S	O-C4	OP-LM	12	100% remained intact; 100% fusion rate
Salunke [22]	2018	6 (6/0)	56	-	AAD posterior (6), OF A-D IIb	S	C1-C2 (6)	LM-P (6)	22	16.7% remained intact; 83.3% improved; 100% fusion rate
Ma [23]	2018	10 (6/4)	50	N/A	AAD (10); OF (10)	S	C1-C2 (6), O-C2 (2), O-C3 (1), C1-C4 (1)	LM-P (7)	22.2	100% improved; 100% fusion rate
Abouelleil [24]	2018	1 (0/1)	19	RTA	AOD, AAD	S	O-C3	OP-LM-P	12	100% remained intact and NP reduced
Kumar [25]	2018	1 (1/0)	11	RTA	AAD (spondyloptosis), OF	S	C1-C2	LM-P	6	100% improved
Robles [26]	2018	1 (1/0)	67	Swimming	AOD rotatory, OCF type III	S	O-C1	N/A	4	100% remained intact and NP reduced
Anania [27]	2018	1 (1/0)	74	Fall	AOD incomplete, AAD anterior; OCF	S	O-C4	OP-LM-SLW-TAS	N/A	100% remained intact
Eghbal [28]	2018	1 (1/0)	21	RTA	AAD rotatory	S	C1-C2	LM-P	6	100% improved and NP reduced
Russo [29]	2017	1 (1/0)	22	RTA	AAD vertical, OCF type II, spinal hygroma	S	C1-C2	LM-TLS	12	100% improved; 100% fusion rate
Nowell [30]	2017	1 (1/0)	71	RTA	AAD posterior, bilateral VA occlusion, C1 Gehweiler I	S	C1-C2	LM-P	N/A	100% remained intact
Larsen [31]	2017	1 (0/1)	75	Fall	AAD rotatory	S	C1-C2	SLW-TLS	6	100% remained intact and NP reduced; 0% fusion
Wang [3]	2017	18 (15/3)	39,2	RTA (11), fall (5) direct hit (2)	AAD (18); OF (13), JF (1), HF (4)	S	C1-C2 (12), OC (6)	N/A	15,3	52.94% improved; 47.06% stable; 83.3% fusion
Hale [32]	2017	1 (0/1)	1	RTA	AOD vertical; AAD vertical	S	O-C2	OP-LM-P	12	100% improved; 100% fusion rate
Eghbal [33]	2017	1 (1/0)	35	Fall	AAD rotatory Fielding I	S	C1-C2	LM-P	N/A	100% improved
Ivetic [34]	2017	1 (1/0)	54	Sports	AAD anterior	S	C1	LM-TLS	N/A	100% improved and NP reduced
Peyriere [35]	2017	5 (2/3)	60	RTA (1), fall (3), epilepsy (1)	AAD rotatory (5)	S	C1-C2 (2), C1-C3 (3),	LM-P (4), TAS (1)	12	80% remained intact, 20% stable; 100% NP reduced and fusion rate
He [36]	2016	1 (1/0)	72	Fall (1)	AAD posterolateral; OF A-D II	S	C1-C3	LM-P	12	100% stable and persistent NP; 100% fusion rate
Han [37]	2016	1 (0/1)	19	RTA	AAD rotatory; HF III	C	Nonoperative	Bidirectional traction for 3 w	3	100% stable and NP reduced
Ueda [38]	2016	1 (1/0)	57	Bicycle fall	AOD posterior; OCF, contusion of cerebellar hemisphere	S	O-C3	OP-LM-P	60	100% improved and NP reduced; 100% fusion rate
Dahdaleh [39]	2016	6 (4/2)	33,3	N/A	AOD (6), OCF (1), JF (1)	S	O-C2 (1), O-C3 (4), O-C4 (1)	OP-LM-P (6)	15,4	66.7% improved, 16.7% remained intact, 16.7% deteriorated and died
Walbom [40]	2016	1 (0/1)	6	Fall	AAD rotatory and displaced ossiculum terminale	C	Nonoperative	Halo traction for 21 w	26	100% improved and NP reduced
Beez [41]	2016	1 (1/0)	9	RTA	AOD	S	O-C3	OP-P	12	100% improved
Mendenhall [42]	2015	23 (14/9)	36,9	RTA (23)	AOD (23): anterior (10), posterior (9), distractive (3)	S	O-C2 (7), C1-C2 (2), O-C3 (9), O-C4 (4), O-C5 (1)	N/A	3	21.74% improved, 73.91% stable; 4.35% deteriorated
Krishnan [43]	2015	1 (1/0)	10	Fall	AAD rotatory	S	C1-C2	LM-P	N/A	100% improved; pain stable
Salunke [44]	2015	6 (N/A)	N/A	RTA (5), fall (1)	AAD anterior (5), posterior (1); OF (6)	S	C1-C2 (6)	LM-P (6)	14	33.3% remained intact; 66.6% improved; 100% NP reduced; 83.3% fusion rate
Hawi [45]	2015	1 (0/1)	34	RTA	AAD rotatory	C	Nonoperative	Halo traction 2 w, rigid collar 6 w	6	100% remained intact and NP reduced
Yang [46]	2015	1 (1/0)	70	RTA	AAD rotatory	C	Nonoperative	Traction, rigid collar 8 w	6	100% remained intact % NP reduced
Xu [47]	2015	1 (1/0)	54	RTA	AAD posterior F	S	C1-C2	AEO + LM-P	12	100% improved and NP reduced; 100% fusion rate
Hu [48]	2015	1 (1/0)	50	Fall	AAD posterior	S	C1-C2	TOO + LM-P	15	100% remained intact and NP reduced; 100% fusion rate
Salunke [49]	2015	4 (4/0)	41	N/A	AAD posterior (1), anterior (3)	S	C1-C2	LM-P	13	100% improved and NP reduced; 100% fusion rate
Chaudhary [50]	2015	1 (1/0)	26	RTA	AAD rotatory and HF L-E IIa	S	C1-C3	LM-P-SLW	16	100% remained intact; 100% fusion rate

^a^C for conservative treatment; S for surgical treatment

^b^Terms intact/improved/stable/deteriorated apply to neurological status

AEO, anterior endoscopic odontoidectomy; A-M, Anderson-Montesano classification; FU, follow-up; OO, os odontoideum; OF, odontoid fracture; OCF, occipital condyle fracture; w, weeks; mo, months; n, number of cases; NP, neck pain; SLW, sublaminar wire; OP, occipital plate; RTA, road traffic accident; TAS, transarticular screw; TLS, translaminar screw; LM, lateral mass screw; TOO, transoral odontoidectomy; JF, Jefferson fracture; N/A, not available or not specified

**Table 4:** Multiple linear regression model to determine factors associated with neurological status at follow-up visits in patients with craniovertebral junction dislocation

	Neurological status at follow-up	
Variable	*β*	*p*
Age at operation	− 0.08	0.514
Sex (F/M)	0.19	0.120
Type of dislocation (AOD/AAD)	0.12	0.307
Plating the occiput (no/yes)	− 0.30	*0.023*

## Discussion

In this study, we touched upon several important aspects. As Winegar et al. in 2010 noted, neck pain improvement after treatment of dislocation of CVJ is generally highest in traumatic cohort [51]. They had detailed information on pain in 28 post-traumatic cases, and improvement after treatment was seen in 22 (78.57%). Our review gathered data on pain from 43 patients with improvement of 83.7% (*n* = 36), confirming a high rate of pain alleviation in this group. According to the literature, after cervical injury, over 60% of survivors experience pain within the first 6 h, over 90% after a day, and basically everyone after 3 days following the trauma [52]. Fortunately, as we presented, successful stabilization paired with further rehabilitation can mitigate it better than in cases of other CVJ abnormalities. Importantly, as Debernardi noticed, if neck pain and stiffness are mild to moderate, it is more likely to overlook the diagnosis [53].

Atlantooccipital joint is regarded as the most stable joint of the body, whereas atlantoaxial joint as the most mobile one [1, 54]. Mobility of the latter, however, is the source of its susceptibility to dislocation. Hence, most cases of the CVJ dislocation happen at the atlantoaxial level. Atul Goel is convinced that majority of AAD do not require involvement of the occiput because there is no concurrent AOD [55]. He states that primary AAD needs only segmental fixation. Extensive multiple-level stabilization compromises the strength of the fusion. In addition, immobilization of both crucial units considerably reduces range of motion. Nevertheless, we showed that 18.2% of patients with pure AAD (*n* = 16) had occipitocervical stabilization. This might be even of higher importance considering the fact that in the presented analysis, involvement of the occipital bone was associated with lower chance of neurological improvement and higher risk of neurological stagnation, especially in cases of pure AAD without AOD. These findings, however, require confirmation in larger prospective studies. Authors of this review generally accept Goel’s attitude that occipitocervical fusion is appropriate and indicated in confirmed atlantooccipital dislocation. Good radiological markers for combined AAD and AOD facilitating decision-making seem to be basion-dental interval (BDI), revised C1-condyle interval (rCCI), and basion-axial interval (BAI) [56]. Especially rCCI with cut-off value of 2.5 mm has high sensitivity and specificity [56].

Our analysis revealed that a method of fusion which was chosen most often was Goel-Harms technique. C1-C2 segment was targeted in 61 patients. Of them, 49 had their construct details reported. Thirty-seven patients (75.5%) had implanted screws/rod construct in accordance with Goel-Harms approach. Rarely did authors from the selected studies choose wiring, and if so, it was mostly in children. From historical perspective, wiring was among the first of techniques introduced to the CVJ battlefield. Although wiring has shortest time to fusion, its failure rate is higher than that of Goel-Harms’ rod-screw construct passing through the lateral masses of C1 and the pedicles of C2 [51].

In 1994, Goel introduced a concept of placing screws into both C1 lateral masses and C2 pedicles [57]. His initial idea back then was to put it monocortically. It was not until 2001 when Harms published his work stressing that bicortical purchase was desired [58]. Harms improved Goel’s approach by applying polyaxial screws bicortically which provided stability for fully loaded rod connection. Goel-Harms C1-C2 fixation has several advantages over the classic Magerl’s transarticular technique. First of all, it does not sacrifice the facets of the first two vertebrae. Omitting disruption of the capsule renders it potentially reversible which might save the range of motion after removal of the construct once the fracture has healed. Also, an angle required to perform the Goel-Harms stabilization is significantly easier to achieve since it is only ~ 22 degrees cephalad when compared with ~ 50 degrees of the Magerl [59–61]. Finally, the Goel-Harms procedure provides a way for one-stage open reduction of the dislocation and fusion utilizing the same anesthesia period and the same positioning [49, 61].

In this paper, sixteen patients (12.4% of the operatively treated) had translaminar screws implanted. This concept was introduced in 2004 by Wright [62]. One of the conditions that needs to be met is transverse diameter of the lamina larger than 3.5 mm [63]. Moreover, C1 lateral mass-C2 laminar method is inferior to Goel-Harms in terms of the stability during lateral bending and axial rotation [64]. It also poses a higher risk of damaging the spinal cord [65]. Additionally, from biomechanical point of view, it is not suitable for longer subaxial constructions when concomitant instabilities are present. However, it can be considered a rescue procedure in situations when C2 pedicle screws cannot be used.

Recently, new techniques began to emerge such as anterior transcervical or transnasal endoscopic odontoidectomy with reduction of the dislocation [47, 66, 67]. There was one such a case in our review. Notably, it is helpful in posterior atlantoaxial dislocation without associated dental fracture when the dens moves anteriorly and is challenging to reduce otherwise. Odontoidectomy might also be performed in a scarless fashion. These are, however, limited options for traumatic patients because of frequent concomitant nasofacial injuries as the most common mechanism of CVJ dislocation is still high-speed road traffic accident.

Our review sheds light upon the lack of unison in terms of methodology of reporting traumatic craniocervical junction sequelae. Too many potentially contributory papers had to be excluded because of insufficient data. Post-traumatic patients are often not distinguished from the reported cohort because of their relatively small number. It is understood that merging etiologies of the CVJ dislocation produces larger series, but it is at a cost of heterogeneity. One solution to this might be providing more details on the subgroups within the mixed series. Furthermore, for case reports as well as case series, it is recommended to furnish detailed and quantified neurological examination done prior to surgery and thereafter. Additional assessment at a distant follow-up would also be of high informative value. Obviously, for part of post-traumatic patients, preoperative evaluation is very challenging due to their poor general state. Those with delayed presentation, however, could be examined thoroughly with at least one of the following scales: mJOA, JOA, ASIA, or Nurick.

## Limitations

First limitation of this research is absence of evidence stronger than level IV. Most authors report their cases in small series, rarely with any control group. Therefore, a meta-analysis based entirely on case reports/case series was carried out. This might bias the scientific truth because there is a tendency to present rather positive outcomes in case reports. Another restraint might be a scope of years of the included papers. We believe, however, that some relevant progress had been made in the field of resuscitation, diagnostic tools, and neurosurgical management of the craniovertebral junction dislocation; therefore, only new publications from the last half a decade were accepted. Finally, lack of unanimous agreement on the manner in which outcomes are reported renders the outcome variable categorical rather than continuous.

## Conclusion

Traumatic dislocation of CVJ is no longer equivalent to death. Due to advances in pre-hospital care, it has become a survivable injury. In-hospital management of choice is timely reduction, at first close with monitoring the patient’s status. In case of irreducible dislocation, open reduction is mandatory with subsequent stabilization. For atlantoaxial dislocation with no other abnormalities of the cervical vertebrae, posterior C1-C2 fusion with the Goel-Harms method is the most appreciated approach, currently regarded as the gold standard. For atlantooccipital dislocation, occipitocervical instrumentation yields satisfactory results. For combined AAD and AOD, superior extension for Goel-Harms technique towards the occiput by means of occipital plating is recommended. Many survivors remain with no deficits or improve, rarely deteriorate. Involving the occiput, especially for pure AAD, might be related with hindered neurological improvement.

## Electronic supplementary material

ESM 1 (DOCX 19 kb).

ESM 2 (DOCX 13 kb).
