# Supplementary material for: Management of post-traumatic craniovertebral junction dislocation: A PRISMA-compliant systematic review and meta-analysis of casereports
Source: Neurosurg Rev. 2020 Aug 14;44(3):1391–400. doi: 10.1007/s10143-020-01366-4 (PMC8121741; doi:10.1007/s10143-020-01366-4)
Supplement: Supplementary file 2 — (DOCX 13 kb). [file 10143_2020_1366_MOESM2_ESM.docx]

**Electronic Supplementary Material No. 2**

Full search strategy

PubMed MEDLINE – searched at 17^th^ February 2020.

(((((((((traumatic atlantoaxial instability) OR traumatic atlantoaxial dislocation) OR traumatic atlantooccipital instability) OR traumatic atlantooccipital dislocation) OR traumatic c1-c2 dislocation) OR traumatic craniovertebral dislocation) OR traumatic craniovertebral instability) OR craniovertebral dislocation) OR craniocervical dislocation) OR craniocervical instability

Web of Science – searched at 19^th^ February 2020

traumatic atlantoaxial instability OR traumatic atlantoaxial dislocation OR traumatic atlantooccipital instability OR traumatic atlantooccipital dislocation

This material is part of the manuscript entitled ‘Management of post-traumatic craniovertebral junction dislocation: A PRISMA-compliant systematic review and meta-analysis of case reports’ authored by
T. Klepinowski, B. Limanówka, L. Sagan.

Corresponding author: T. Klepinowski

Affiliation: Department of Neurosurgery, Pomeranian Medical University Hospital No 1, Szczecin, Poland

E-mail address: [tomasz.klepinowski@pum.edu.pl](mailto:tomasz.klepinowski@pum.edu.pl)
